# First isolation of rabies virus from a Eurasian badger (*Meles meles*) in Inner Mongolia, China, 2024

**DOI:** 10.3389/fvets.2025.1598528

**Published:** 2025-06-25

**Authors:** Sixu Chen, Anyu Bao, Gerile Aodun, Pei Zhang, Lu Zhang, Nan Gao, Honglong Qiao, Wenying Liu, Qiang Liu, Yufei Zhang

**Affiliations:** ^1^College of Veterinary Medicine, Inner Mongolia Agricultural University, Hohhot, China; ^2^Wildlife Conservation Society of the Inner Mongolia Autonomous Region, Hohhot, China; ^3^School of Life Sciences, Inner Mongolia University, Hohhot, China; ^4^Forestry and Grassland Bureau of Qingshuihe County, Hohhot, China; ^5^Urban Management and Law Enforcement of Qingshuihe County, Hohhot, China; ^6^Hohhot Landscape Construction Service Center, Hohhot, China

**Keywords:** rabies virus, virus isolation, Eurasian badger, public health, livestock, phylogenetic analysis, zoonotic disease

## Abstract

Rabies continues to pose a significant global zoonotic threat. In recent years, the increased spillover events of rabies viruses from wildlife to domestic animals have raised public health security concerns, prompting heightened international attention toward rabies management in wildlife populations. Our study reveals the first documented case of a rabies virus (RABV) strain isolated from Eurasian badgers (*Meles meles*) within Chinese ecosystems. Genetic analysis shows 99.4% nucleotide identity with dominant bovine-associated cosmopolitan lineages, offering robust evidence of interspecies transmission from wildlife reservoirs to domestic livestock. It is noteworthy that due to the special geographical location of this region, the habitat of Eurasian badgers overlaps with the territory of livestock and human settlements, thereby forming a transmission chain of rabies virus such as “fox- Eurasian badger-livestock” or “Eurasian badger-livestock.” This critical finding highlights an urgent need for enhanced pathogen surveillance programs in pastoral regions where intensive human-wildlife-livestock interfaces create high-risk transmission zones.

## 1 Introduction

Rabies, caused by the neurotropic rabies virus (RABV), is an invariably fatal zoonotic disease and a global public health concern (1). Despite extensive control measures, RABV continues to circulate worldwide, resulting in approximately 59,000 human deaths annually (2). Historically, the primary reservoirs of RABV were dogs; however, this pathogen persists in Latin America within sylvatic cycles through terrestrial and airborne species (3). In the United States, canine rabies was eliminated in the 1970s following rigorous national control efforts in the early 1940s. Today, approximately 60,000 Americans receive post-exposure prophylaxis for rabies annually, primarily due to interactions with wildlife and unvaccinated domestic animals (4). This highlights the public health risks posed by wildlife through the direct transmission of RABV. Currently identified wildlife reservoirs include bats, raccoons, skunks, foxes, and wolves (5). The degree of viral adaptation to these species remains challenging due to limited evidence. However, viral variants predominantly circulate within specific reservoir hosts, particularly among medium- and small-sized carnivores (5). Strengthening rabies surveillance in these wildlife species is crucial for informing vaccine development and optimization as well as mitigating potential outbreaks.

Phylogenetic analyses classify RABV into five major lineages: African, Asian, Cosmopolitan, Arctic-related, and Indian (6). As the nomenclature suggests, the virus exhibits distinct geographical distribution patterns. The Asian lineage is restricted to Southeast Asia, while Arctic- and the Indian subcontinent-related lineages have been unexpectedly detected in South Australia (6). In China, the cosmopolitan lineage remains dominant, while the Arctic-related (AL2) and Asian (SEA1) lineages are rarely reported (7). Since 2013, northern China has documented rabies cases in both domestic animals, including cattle, sheep, and camels, as well as in wild carnivores such as wolves and foxes (7–10).

Ecologically, mesopredators such as foxes, which occupy intermediate trophic levels as both predators and prey, significantly contribute to RABV transmission. In China, foxes have emerged as the second most important rabies reservoir, primarily transmitting cosmopolitan RABV strains (7). This finding highlights the crucial role of medium-sized carnivores in rabies epidemiology. Mustelids also occupy similar ecological niches to foxes in food webs. Globally, rabies infections have been reported in ferret badgers (*Melogale moschata*) (11) and honey badgers (*Mellivora capensis*) (12). Unlike the typically timid nature of foxes, mustelids display pronounced aggression and combativeness, potentially increasing their rabies transmission capacity. Notably, the Eurasian badger (*Meles meles*), a widely distributed mustelid across Eurasia, has been identified as a rabies virus reservoir in Europe (13), then no Eurasian badger rabies cases have been reported in China.

In this study, we confirmed a rabies-positive Eurasian badger case through comprehensive diagnostic approaches, including histopathological examination, laboratory testing, and virus isolation. To facilitate timely data dissemination, RABV was isolated from badger brain tissue, whole genome sequencing was performed, and the complete genome sequence was submitted to the NCBI database. Additionally, a comparative phylogenetic analysis was conducted to determine viral origins and lineage classification. This study aims to confirm the first documented case of rabies virus infection in Eurasian badgers (*Meles meles*) in China, characterize the viral strain through genomic and phylogenetic analyses, and evaluate its implications for regional rabies ecology and public health.

## 2 Methods

### 2.1 Sample collection

In April 2024, within the administrative boundaries of Qingshuihe County (40°16′N, 111°40′E), Hohhot City, Inner Mongolia Autonomous Region, China, Eurasian badgers encroached into human settlements and displayed aggression toward residents. On April 11, a badger attacked a flock of ovines belonging to a shepherd. The shepherd subdued and captured the badger, subsequently reporting the incident to the Wildlife Conservation Society of the Inner Mongolia Autonomous Region. The badger died within 24 h, and its carcass was sent to the Microbiology Laboratory of the College of Veterinary Medicine, Inner Mongolia Agricultural University, for dissection.

### 2.2 Case identification

During routine necropsy, brain tissues were fixed in 10% neutral-buffered formalin, while residual brain tissue was stored at −80°C for future analyses. Fixed tissues were paraffin-embedded, sectioned, and stained with hematoxylin and eosin (H&E) for histopathological evaluation. Total RNA was extracted from brain tissue according to the instructions of the EasyPure® Viral DNA/RNA Kit (TransGen Biotech Co., Ltd., Beijing). RNA samples were resuspended in 50 μL of RNase-free water and stored at −80°C for short-term preservation prior to downstream analyses. The obtained viral RNA was reverse-transcribed using a one-step RT-PCR kit (Vazyme Biotech Co., Ltd., Nanjing), following the manufacturer’s instructions. PCR amplification targeted the N gene region using rabies primers recommended by the World Organisation for Animal Health (WOAH, formerly OIE). To validate the reliability of our assays, we incorporated the Negative control to confirm no cross-contamination. Finally, the gel was visualized under UV light using the AIIDoC-x system (Tanon).

For immunohistochemical analysis, 4 μm paraffin sections were dewaxed with graded ethanol (75%) and rehydrated. Antigen retrieval was performed using 0.1% trypsin solution. Following the mouse/rabbit universal SAP kit protocol (Zhongshan Jinqiao Biotechnology Co., Ltd., Beijing), endogenous peroxidase activity was blocked, and a goat serum blocking solution (Thermo Fisher Scientific-CN) was applied. Sections were incubated overnight at 4°C with rabbit anti-RABV phosphoprotein (P) polyclonal antibodies (kindly provided by Prof. Ling Zhao, Huazhong Agricultural University), followed by phosphate-buffered saline (PBS) washes and sequential application of biotin-labeled goat anti-rabbit IgG and horseradish peroxidase (HRP)-labeled streptavidin working solution (Thermo Fisher Scientific-CN). PBS replaced the primary antibody in negative controls. Visualization was performed using 3,3′-diaminobenzidine (DAB) chromogen and sections were mounted in neutral resin for microscopic examination.

### 2.3 Virus isolation and TEM imaging

Brain homogenates were centrifuged (3,000 × g, 15 min), filtered (0.22 μm), and inoculated into BHK-21 cells (ATCC CCL-10) for blind virus passage. Following each collection of cell supernatant, viral RNA was extracted from the cell supernatant, and the same PCR identification method as that used for brain tissue was used to determine whether the virus continued to multiply. The cell culture supernatant was discarded, and cells were PBS-washed three times. The BHK-21 cells were fixed in methanol/acetone (1:1) at −20°C for 20 min, blocked with 5% skim milk (500 μL), and subsequently incubated with FITC-conjugated anti-RABV P protein antibodies (1:200 dilution, provided by Prof. Ling Zhao) at 37°C for 1 h. Following PBS washes, nuclei were counterstained with DAPI (1:1000) at 37°C for 15 min before examination via confocal microscopy. Following three blind passages,

For transmission electron microscopy (TEM), BHK-21 cells were fixed in 2.5% glutaraldehyde (0.1 M phosphate buffer, pH 7.4) at 4°C for 2 h, followed by post-fixation in 1% osmium tetroxide/1% potassium ferrocyanide at 25°C for 1 h. Samples underwent a graded ethanol dehydration series (50–100%) and were embedded in epoxy resin. Ultra-thin sections (60 nm) were prepared using a Leica EM UC7 ultramicrotome and stained with uranyl acetate/lead citrate. Imaging was conducted using a Hitachi H-7650 TEM.

### 2.4 Full-genome sequencing and phylogenetic analyses

Full-genome primers were designed based on published RABV sequences (Table 1). cDNA synthesized in section 2.2 served as the PCR template. Amplicons were gel-purified and subjected to commercial sequencing. These sequences were assembled and edited using SeqMan (DNAStar 5.0) and then submitted to NCBI. Sequence alignments and phylogenetic reconstruction were conducted using DNAStar v7.2 and MEGAX, employing the maximum likelihood method with 1,000 bootstrap replicates.

**Table 1:** Primers for full sequence amplification of rabies virus.

Primer name	Sequence 5′-3′	Length (bp)
3-RA-F-1	TACGCTTTAACCACAAATCAGAG	530
3-RA-R-530	TATTTTGCTCAACCTATACAGACTC	
RA-F-20	GAGAAGAAGCAGACAGCGTCA	1,544
RA-R-1564	TCTCTTCAGCCATCTCAAGATCG	
RA-F-1271	AAGTCCAGAAGCCGTTTATACTC	1,471
RA-R-2741	TCTCGTCAAATGATCTCAAAATG	
RA-F-2511	CAAGATAGTGAAAAACTGTAGGGAT	1,860
RA-R-4371	GGACTGACTTGTAGTGAGCATC	
RA-F-4141	GTTGGTGAATCTGCACGAC	1,679
RA-R-5820	CGTACAATTTAACAGCTTCTCTA	
RA-F-5491	CCTAACATCTTGAGAAACTCTGACT	1,649
RA-R-7140	CAAGATGTAGTTAGCCAGGAGC	
RA-F-7061	GTCGATTCTTTGCTCTAATGTCA	1,519
RA-R-8580	AAACTGCCTTCTTATCGTCCG	
RA-F-8421	GTTGTCCAAGACCCATAGAGATAA	1,790
RA-R-10211	TCCGCAGAGAGTCTTTTAGTAGTC	
RA-F-10091	GTATGAGAGCTAGCCTGCGAC	1,700
RA-R-11791	CAAGCAGCTGTAGTCCAGTAGAG	
5-RA-F-11411	GGCACTTCAACATTTGTTGCA	496
5-RA-R-11907	CGCTTAACAAAAAAWCMAC	

## 3 Results

**Figure 1:**
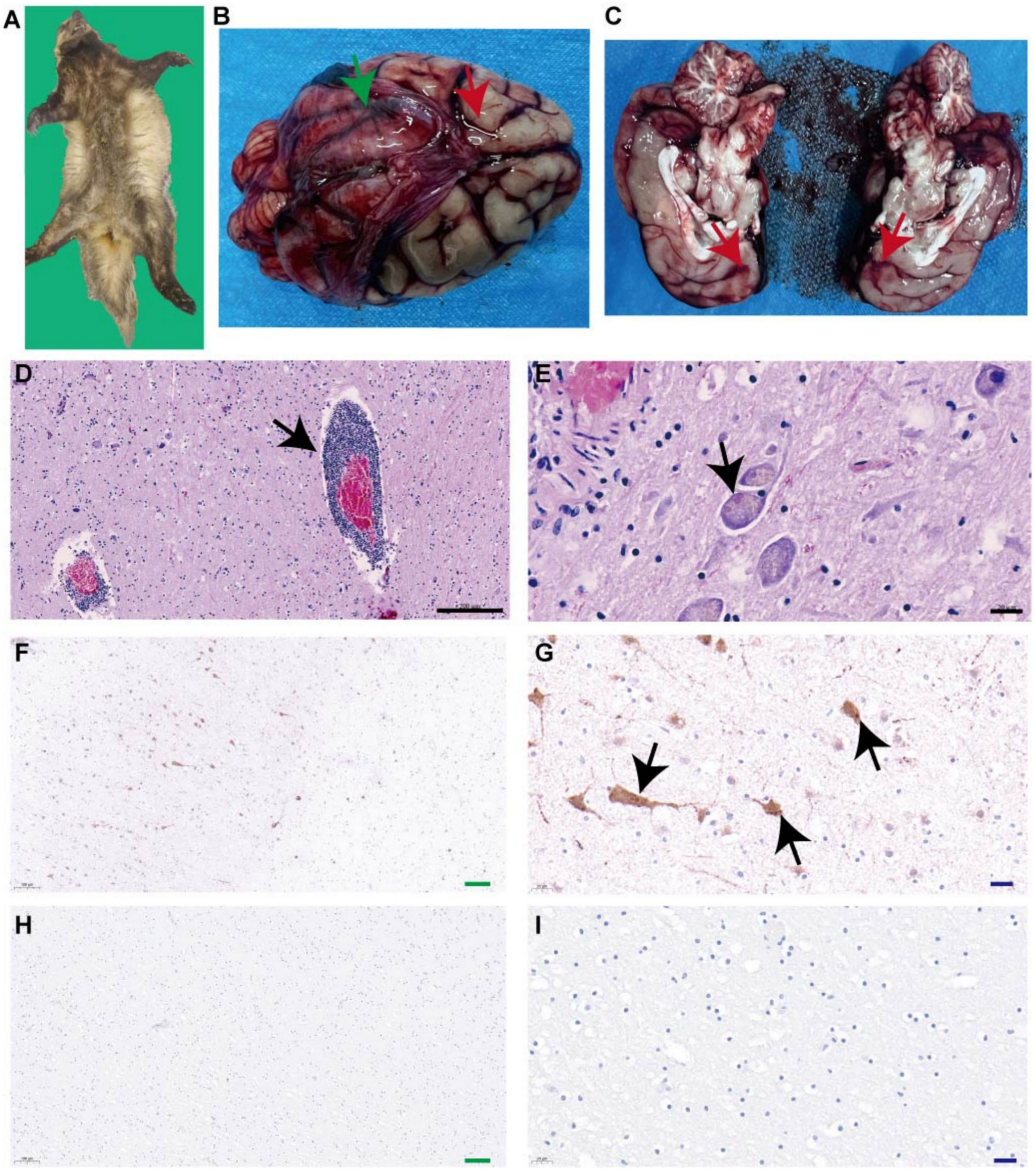
Identification of rabies virus in Eurasian badger. **(A)** Eurasian Badger carcasses. **(B)** Green arrows indicate congestion in brain tissue, and red arrows indicate cerebrospinal fluid accumulation. **(C)** Red arrows indicate scattered punctate hemorrhages in the brain parenchyma. **(D)** Black arrows indicate demonstrated perivascular cuffing by lymphocytes and macrophages. **(E)** Black arrows indicate eosinophilic Negri bodies in neuronal cytoplasm. **(F,G)** Black arrows indicate brownish-yellow positive signals. **(H,I)** Phosphate-buffered saline was used as the primary antibody, and the results showed no obvious brown positive signal.

Postmortem examination of the Eurasian badger (*Meles meles*) carcass revealed no external trauma or signs of physical injury (Figure 1A). Postmortem examination revealed meningeal congestion with cerebrospinal fluid accumulation (Figure 1B) and scattered punctate hemorrhages in the brain parenchyma (Figure 1C). No external injuries or visceral abnormalities were observed.

**Figure 2:**
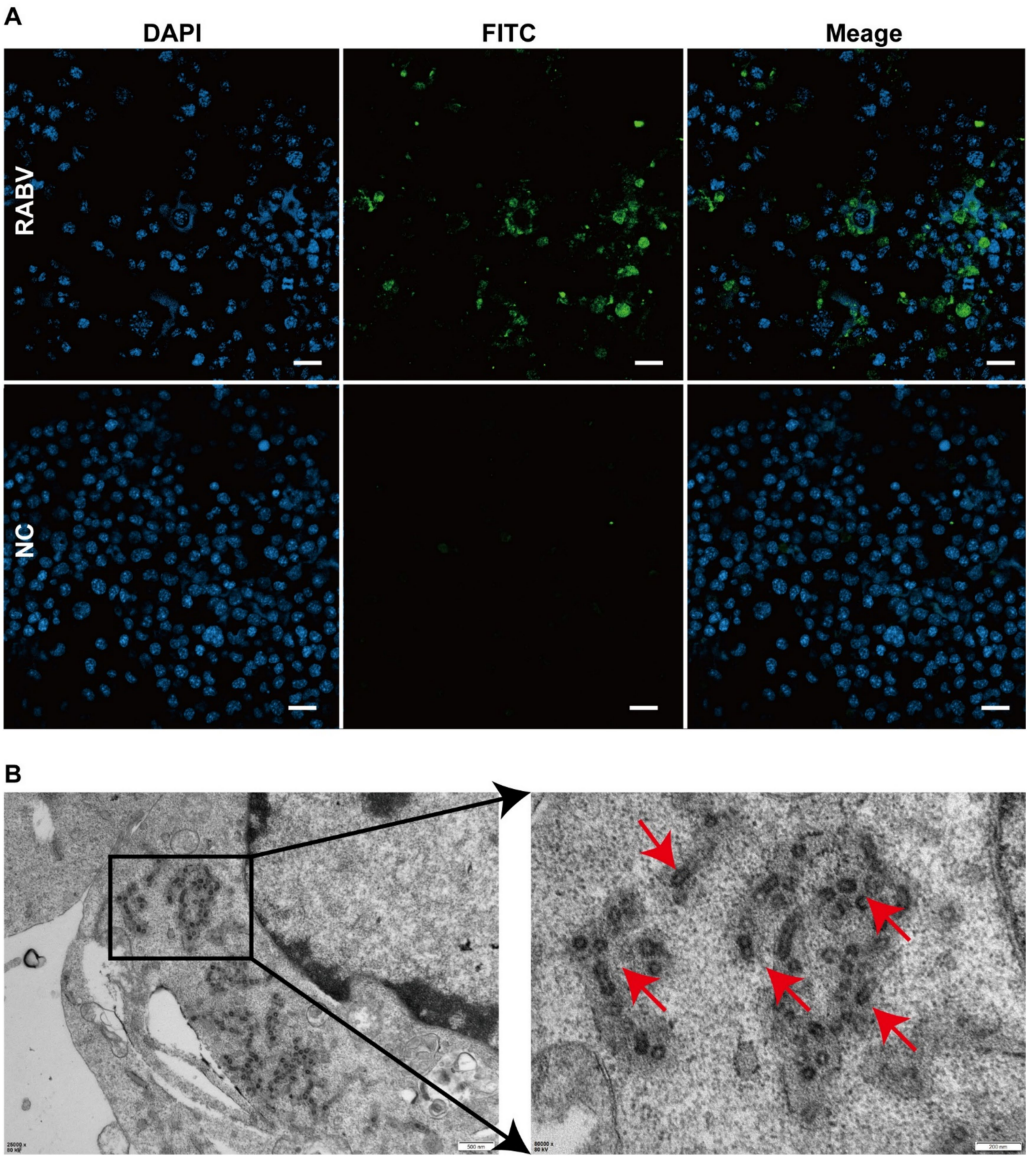
Immunofluorescence analysis and Rabies Virus Isolation. **(A)** Green fluorescence indicates virus-infected cells. No fluorescence was detected in the control group. **(B)** Red arrows indicate rabies virus particles.

A specific band was obtained at the expected size of approximately 500 base pairs (bp), suggesting that the badger was infected with rabies. Hematoxylin–eosin staining demonstrated perivascular cuffing by lymphocytes and macrophages (Figure 1D) and eosinophilic Negri bodies in neuronal cytoplasm (Figure 1E). Specific brown cytoplasmic staining confirmed the presence of the RABV antigen in hippocampal neurons (Figures 1F,G), while control sections showed no signal (Figures 1H,I). After three blind passages, viral replication was confirmed using FITC-conjugated anti-RABV P protein antibodies (Figure 2A). Negative staining transmission electron microscopy revealed bullet-shaped virions measuring 120–220 nm in length and 50–60 nm in width (Figures 2B), consistent with RABV morphology.

**Figure 3:**
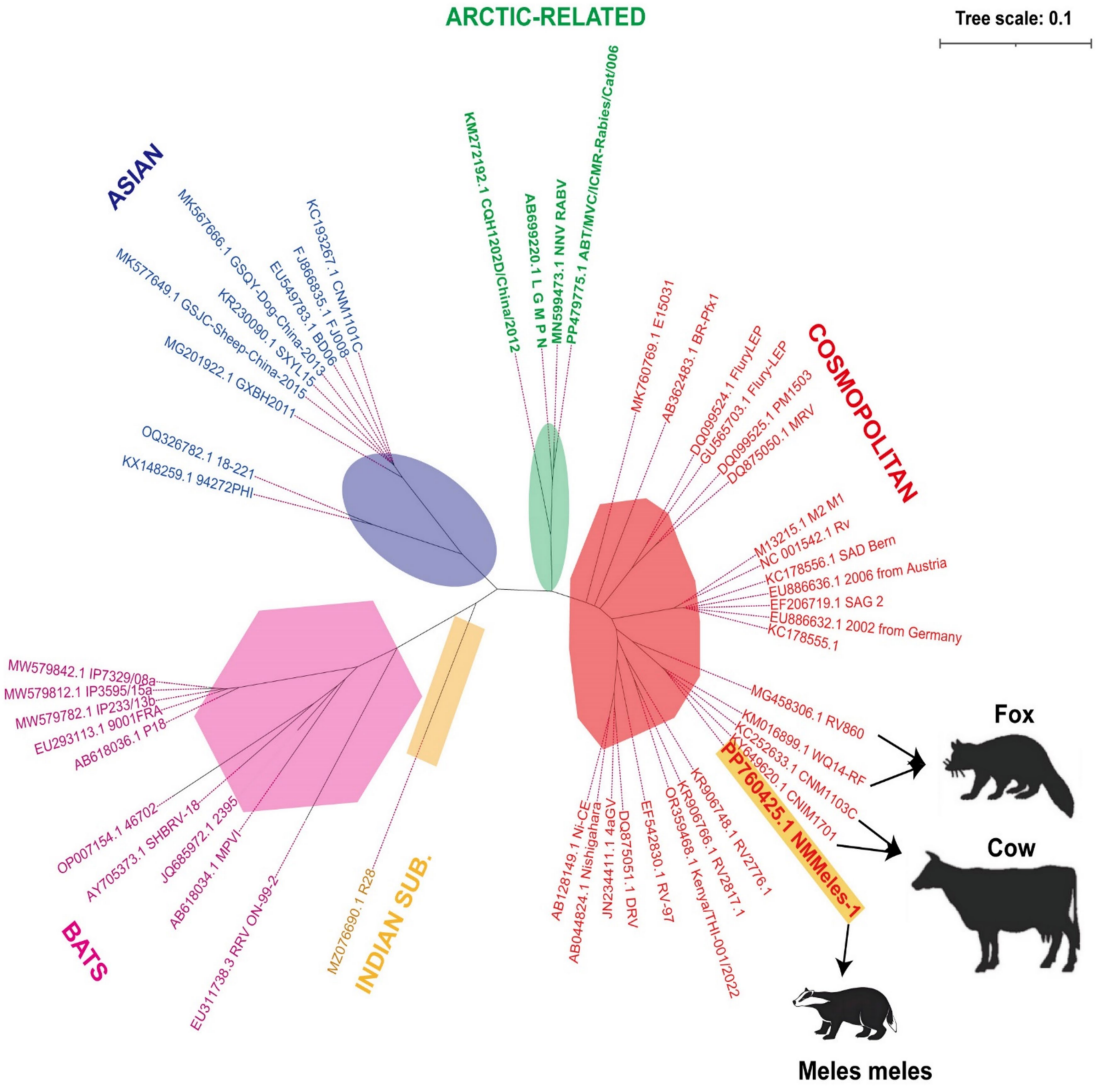
Phylogenetic analysis of the full-length genome sequence of NMMeles-1. Yellow highlighting indicates rabies virus sequences isolated from Eurasian Badger. Black arrow indicates the host of the rabies virus. Background colors indicate viruses belonging to the same coronavirus genus. Scale bar represents 0.1 substitutions per site.

Whole-genome sequencing (Illumina NovaSeq 6000) generated an 11,932-nucleotide sequence (GenBank: PP760425; strain NMMeles-1). In a phylogenetic analysis using the maximum-likelihood method with 1,000 bootstrap replicates implemented in MEGA X, NMMeles-1 was assigned to the cosmopolitan lineage, showing 99.4% identity with bovine strain CNM1103C from Inner Mongolia (Figure 3).

## 4 Discussion

Rabies is primarily transmitted through bites (14). However, the badger carcass showed no visible wounds. This suggests a prolonged viral latency, potentially establishing badgers as reservoir hosts, similar to foxes. Histopathological examination of a rabies-infected ferret badger revealed perivascular cuffing, characterized by dense infiltration of lymphocytes, plasma cells, and macrophages (15). These findings were consistent with those from histopathological examinations of badgers. Unlike camel rabies, where Negri bodies localize in Purkinje cells (16), eosinophilic Negri bodies were observed within neuronal cytoplasm. Although Negri body remains a diagnostic marker for rabies, it has not been found in some confirmed cases of rabies (16, 17), which is related to the rabies infection period of the cases (18). In addition, Negri bodies are sometimes confused with Negri-like inclusion bodies, which are indistinguishable from each other (19). Therefore, necessitates complementary diagnostics like immunohistochemistry (IHC) (18). Notably, viral antigen distribution in the brainstem was more widespread than in other specific brain regions. Additionally, distinct interspecies variations were observed in RABV antigen distribution among different animal species. For instance, the hippocampus was identified as the primary detection site in dogs and cats, while the brainstem was the main detection site in cattle. In raccoons and skunks, RABV antigens were detectable across all brain tissue compartments (20). This aligns with our IHC findings, which revealed scattered positive signals in brain tissues. TEM observations demonstrated that rabies virus particles exhibited bullet-shaped or spherical morphologies, with dimensions ranging between 120–220 × 50–60 nm (21, 22). These characteristics were consistent with the viral particles isolated from badger brain tissues in our study.

Rabies virus strains originating from wildlife remain the primary sources of AL2 and steppe-type variants (23, 24), which aligns with the phylogenetic analysis results of the NMMeles-1 system. Of the 26 livestock rabies cases with known attackers, about 62.5% (25/40) were caused by fox bites, further emphasizing that foxes play an important role as a reservoir in the transmission of animal rabies in north China (9). While fox-mediated human rabies infections remain rare, a human rabies death caused by a fox bite was reported in the Xinjiang Uygur Autonomous Region of China in May 2016 (25). Notably, both foxes and badgers occupy mesopredator positions within the food chain. Badgers may replace foxes as an epidemiological bridge that facilitates the spread of the virus. A substantial surge in rabies transmission from infected wild animals to domestic animals has been observed, particularly in pastoral regions encompassing grasslands and mountainous areas (26, 27). Between 2012 and 2018, Mongolia reported 2,359 animal rabies cases, with livestock cases (1,380, 58%) significantly outnumbering wildlife cases (317, 13.4%) (28), indicating substantial cross-species transmission. NMMeles-1exhibiting 99.4% identity with fox-derived RV860 strain, supporting transboundary transmission (6). Notably, NMMeles-1 also exhibited 99.4% identity with bovine strain CNM1103C and 98.8% identity with Inner Mongolian fox strain WQ14-RF, submitted in 2012 and 2014, respectively (29), indicating viral persistence for >12 years. These findings inform regional vaccination strategies.

This study confirmed for the first time in China the biological evidence of natural rabies virus infection in Eurasian badger. These findings have several critical implications. First, the high genetic similarity (99.4%) between NMMeles-1 and bovine-derived strains, Considering the unique geographical features of this region that lead to overlapping distributions between human settlements and Eurasian badger (*Meles meles*) habitats, combined with numerous documented cases of badger attacks on humans and livestock in the area, we propose that the Eurasian badger may be a neglected spillover host. Second, as intermediate predators, badgers may act as epidemiological bridges, facilitating viral spillover between primary reservoirs, such as foxes (30) and domestic animals. Globally, livestock rabies cases originating from wildlife reservoirs have risen sharply, with Inner Mongolia incurring significant economic losses due to culling and trade restrictions (28, 30).

## 5 Conclusion

This study confirmed for the first time in China the biological evidence of natural rabies virus infection in Eurasian badger (*Meles meles*), enriching the genetic sequence of viral rabies in wild animals. The isolation of a cosmopolitan RABV lineage from a badger highlights the dynamic nature of cross-species transmission within northern China’s grassland ecosystems. These findings necessitate a paradigm shift in surveillance strategies, emphasizing wildlife reservoir monitoring and vaccine development targeting emerging variants. Sustained interdisciplinary collaboration among ecologists, virologists, and public health authorities will be critical for reducing zoonotic risks and safeguarding both economic livelihoods and ecosystem stability in regions characterized by intense human-animal interactions.

## Data Availability

The datasets presented in this study can be found in online repositories. The names of the repository/repositories and accession number(s) can be found at: https://www.ncbi.nlm.nih.gov/genbank/, PP760425.
